# Abbott RealT*ime* HBV Assay Is More Sensitive in Detection of Low Viral Load and Little Impacted by Drug Resistant Mutation in Chronic Hepatitis B Patients under Nucleot(s)ide Analogues Therapy

**DOI:** 10.1371/journal.pone.0101790

**Published:** 2014-07-07

**Authors:** Ming-Lun Yeh, Chung-Feng Huang, Ching-I Huang, Shu-Fen Liu, Hua-Ling Yang, Ming-Yen Hsieh, Jee-Fu Huang, Chia-Yen Dai, Wan-Long Chuang, Ming-Lung Yu

**Affiliations:** 1 Division of Hepatobiliary, Department of Internal Medicine, Kaohsiung Medical University Hospital, Kaohsiung, Taiwan; 2 Graduate Institute of Medicine, College of Medicine, Kaohsiung Medical University, Kaohsiung, Taiwan; 3 Faculty of Internal Medicine, College of Medicine, Kaohsiung Medical University, Kaohsiung, Taiwan; 4 Department of Occupational Medicine, Kaohsiung Municipal Ta-Tung Hospital, Kaohsiung Medical University, Kaohsiung, Taiwan; 5 Department of Internal Medicine, Kaohsiung Municipal Hsiao-Kang Hospital, Kaohsiung Medical University, Kaohsiung, Taiwan; 6 Department of Internal Medicine, Kaohsiung Municipal Ta-Tung Hospital, Kaohsiung Medical University, Kaohsiung, Taiwan; 7 Hepatitis Center, Kaohsiung Medical University Hospital, Kaohsiung Medical University, Kaohsiung, Taiwan; 8 Graduate Institute of Clinical Medicine, College of Medicine, Kaohsiung Medical University, Kaohsiung, Taiwan; University of Athens, Medical School, Greece

## Abstract

**Background:**

Selection of drug-resistant strains may lead to failure of HBV antiviral therapy. There is little information whether there is detection difference in drug resistant mutations between different viral load assays of HBV.

**Objectives:**

This study is aimed to investigate whether there is drug-resistant strains related detection difference between Abbott RealT*im*e HBV (RealT*ime*) and CobasAmpliPrep/CobasTaqMan HBV assays 2.0 (TaqMan).

**Study Design:**

One hundred and thirty-four CHB patients who received HBV anti-viral therapy were enrolled. HBV virological markers were tested 3 months apart regularly. Serum HBV DNA levels were determined using the TaqMan and RealT*ime*. YMDD (rt180M and rt204V) mutation was checked in patients who experienced virologic breakthrough (VBT).

**Results:**

The correlation of HBV DNA observed between the RealT*ime* and TaqMan was good for all 571 samples (R^2^ = 0.797; *P*<0.001). However, the correlation in the 434 samples with HBV DNA level <3 log_10_ IU/ml was not as good as in all samples (R^2^ = 0.457). Overall, 21.5% of samples had a detection difference of ≥1 log_10_ IU/ml with 91.9% of these having HBV DNA level <3 log_10_ IU/ml. Twenty-four patients experiencedVBT. Three of these patients had acquired the YMDD mutation and exhibited discordant viral load results between the two methods tested. In each case, persistent HBV DNA was detected by RealT*ime* and undetectable with TaqMan. Of the patients who experienced a VBT and had acquired YMDD mutation, 4.7% had undetectable HBV DNA by TaqMan while all were detectable with RealT*ime*.

**Conclusions:**

RealT*ime* assay is more sensitive and is little impacted by the development of drug resistant mutation.

## Introduction

Hepatitis B virus (HBV) infection is a global health problem, with an estimated 350–400 million people being chronically infected [1], [2]. HBV is a small partially double stranded DNA virus that replicates through an RNA intermediate. Because the HBV-encoded polymerase is prone to error, the virus evolves quickly under the selection pressure like adaptive immune response and antiviral therapy. The estimated nucleotide substitution rate of HBV is between 1.5×10^−5^ and 1.9×10^−4^ substitutions per site per year [3], [4], [5]. A recent study demonstrated a higher substitution rate of 8.15×10^−4^ substitutions/site/year in the HBV core gene compared to 4.57×10^−4^ substitutions/site/year in the surface gene [6]. The number and position of these mutations may impact the viability of the virus. In a single study of patients with chronic HBV with active viral infection, 20 nucleotide changeshad accumulated between positions 1933–2048 of the core/pre-core region [7]. This indicates that considerable changes may occur in this region without stopping viral replication.

Assays for viral load quantificationwhich target core/pre-coremay be impacted by nucleotide changes if they resided in sites selected for primers and probes [8]. Differences of greater than 1 log_10_ IU/ml for individual samples have been noted between commercial HBV viral load assays noted even when general correlation between the assays were considered good [9]. In our recent published study [10] evaluatingthe correlations between two commercially available HBV viral load assays (Abbott RealTi*m*e HBV assay, hereafter, “RealTi*m*e” and CobasAmpliPrep/CobasTaqMan HBV assay, hereafter, “TaqMan”), very good correlation was observed in untreated HBV-infected patients. However, 8.6% of patients in this study had discordant results of ≥1 log_10_ IU/mlbetween the two assays, especially in patients with viral load of less than 3 log_10_ IU/ml. Recently, a new developed ultra sensitive in-house real-time PCR assay for HBV DNA quantification was reported by Paraskevis et al. [11]. They found that the clinical performance of the ultra sensitive assay was tested similar to the ProcleixUltrio discriminatory HBV test in low-titer samples and the quantitative results by the ultra sensitive assay were also strongly correlated with COBAS TaqMan HBV assay.

In patients with HBV antiviral therapy, selection of drug-resistant strains may lead to failure of HBV antiviral therapy. It occurred in approximately 16% to 43% of the patients treated with lamivudine for 1 to 2 years [12], [13]. The 5-year cumulative incidence of resistance was only 0–1.2% in patients treated with entecavir and tenofovir, the current recommended first-line therapies with high potency and high genetic barrier [14]. Drug resistance is suspected with a rise in HBV viral load above the detection limit of the assay or ≥1 log_10_ IU/ml from nadir. Currently, there is little information about HBV viral load assay concordance in samples harboring drug-resistant mutations.

This present study aimedto investigate the impact of drug-resistant strainson detection difference between RealTi*m*e and TaqMan.

## Patients and Methods

One hundred and thirty-four CHB patients, who had received treatment with an approved nucleot(s)ide analogue (NUC) for at least 3 months from August 2008 to October 2010 at Kaohsiung Medical University Hospital in Taiwan, were enrolled into this prospective study. All patients were seropositive for hepatitis B surface antigen (HBsAg) for more than 6 months. Anti-viral therapy for HBV and agents used for therapy were decided according to the criteria of the Bureau of National Health Insurance of Taiwan and physician's decision. Patients who were co-infected with hepatitis C virus (HCV), human immune-deficiency virus (HIV) or combined with other liver disease were excluded. HBV virological markers were tested at enrollment, and 3 months apart regularly during study period. Hepatitis B e antigen (HBeAg) was detected using commercially available enzyme-linked immunosorbent assay kits (Abbott Laboratories, North Chicago, IL, USA). Serum HBV DNA levels were determined using the TaqMan (CAP/CTM version 2.0, Roche Diagnostics, Indianapolis, IN, USA; dynamic range 20 IU/ml–1.7×10^8^ IU/ml) and *RealTime* (Abbott Laboratories, Abbott Park, IL, USA; dynamic range 10IU/ml–1×10^9^ IU/mL). The samples were simultaneously tested according to the manufacturer's instructions. Samples measured below lower limit of quantification with target detected were set as undetectable in current study.

YMDD (rt180M and rt204V) mutation would be checked in patients who experiencedvirologic breakthrough (VBT), defined as greater than 10 fold increase in serum HBV DNA from nadir or redetection of HBV DNA in serum after its initial disappearance in 2 consecutive measurements, at the time points of enrollment and VBT. YMDD mutation was determined by the Abbott HBV RUO Sequencing assay (Abbott Molecular Inc., Des Plaines, IL). The processing followed by real-time PCR amplification and detection was performed on the integrated Abbott *m2000sp/m2000rt* instrument system (Abbott Molecular Inc.), using the *m2000* HBV Sequencing Version 2.00 software application and HBV RUO Sequencing Application CD-ROM version 2.0 (Abbott Molecular Inc.). Specimen with a minimal volume of 0.8 ml was required for automated processing by the *m 2000sp*. Detection and quantification of the 1023-bp HBV amplification product was then performed with the *m 2000rt*. Four individual sequencing reactions (2 forward and 2 reverse) were performed for each sample to provide bidirectional sequences spanning the rt/pol and *S* gene regions of interest. All cycle sequencing procedures were performed on a GeneAmp PCR System 9700 (Applied Biosystems, Foster City, CA, USA). The cycle sequencing products were generated with the Big Dye Terminator 3.1 Cycle Sequencing kit (Applied Biosystems) and then analyzed by capillary electrophoresis on an Applied Biosystems 3130*xl* Genetic Analyzer (AB 3130*xl*, Applied Biosystems) according to the assay manufacturer's instructions and analyzed with SeqScape Software, version 2.7 (Applied Biosystems).

The study was conducted according to the guidelines of the Declaration of Helsinki andthe principles of Good Clinical Practiceand was approved by the ethic committee of Kaohsiung Medical University Hospital (KMUH-IRB-970125). Written informed consent was obtained from all patients.

### Statistical analysis

Continuous variables were expressed as median and25^th^, 75^th^ percentile. Categorical variables were expressed as numbers and percentage. Correlation analysis was performed using Pearson correlation coefficients and linear regression. Bland–Altman plots were used to represent the degree of agreement. The mean log_10_ paired difference and 95% limits of agreement are indicated in the figures by solid and dotted lines. All tests were two-sided, and *p*<0.05 was considered significant. All analyses were performed using the SPSS ver17.0 statistical package (SPSS, Inc., Chicago, IL, USA).

## Results

**Figure 1:**
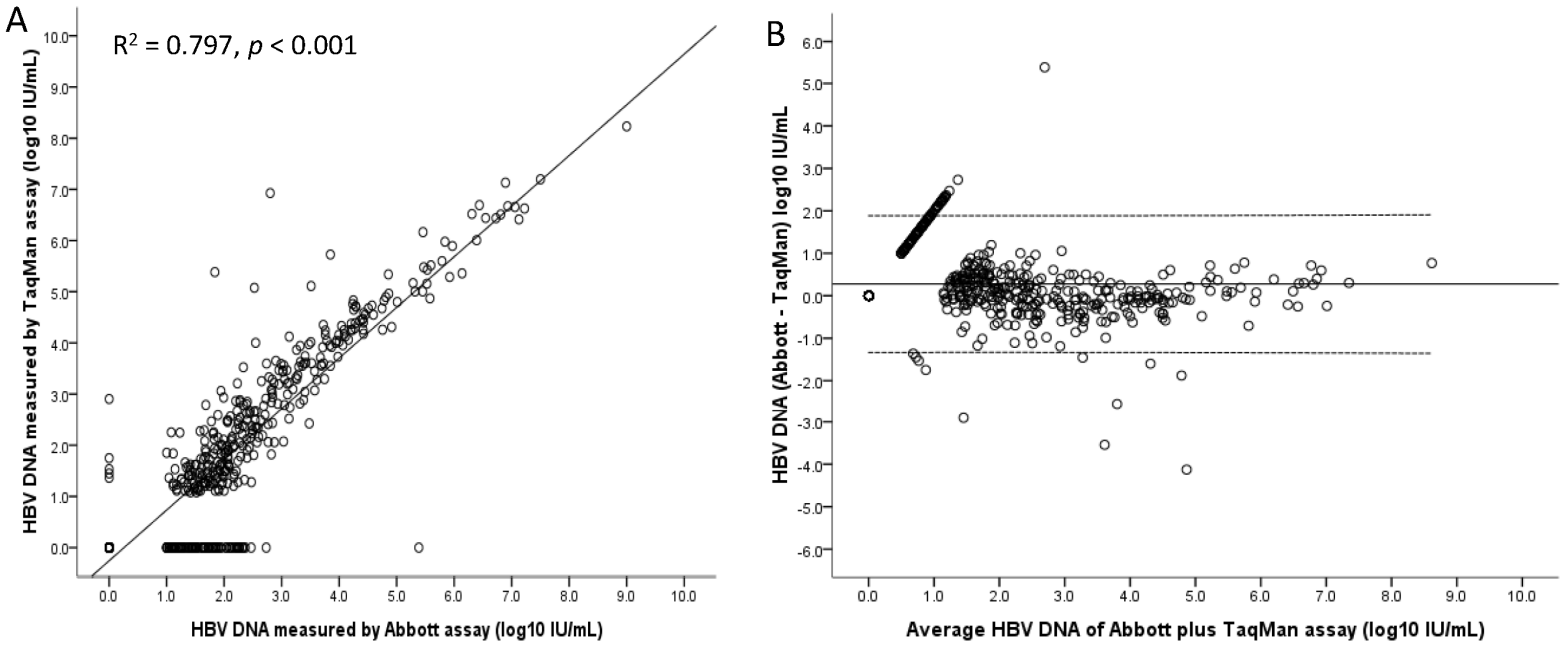
The correlation of measurements and limits of agreement between RealT*ime* and TaqMan assay in all 571 serum samples. The correlation of HBV DNA observed between the *RealTime* and TaqMan assays was good (R^2^ = 0.797; *P*<0.001).(A) The mean difference between the two assays (*RealTime*-TaqMan) was 0.28±0.82 log_10_ IU/ml (limits of agreement, −1.36 to 1.91 log_10_ IU/ml).(B)

Demographics of the 134 CHB patients with antiviral therapy were shown on Table 1. Most of the patients were male with a median age of 45 years old. The median ALT level at enrollment was 34 U/L. The median time from treatment initiation to enrollment was 9 months and 67.2%, 13.4% and 19.4% of patients received lamivudine, telbivudine and entecavir treatment. HBeAg was positive in 45.1% of patients. The measured median HBV DNA level was 1.7 log_10_ IU/ml by TaqMan and 1.9 log_10_ IU/ml by RealT*ime*. Overall, 571 serum samples were obtained during study period and the correlation of HBV DNA observed between the RealT*ime* and TaqMan was good (R^2^ = 0.797; *P*<0.001)(Figure 1A). The mean difference between the two assays (RealT*ime*-TaqMan) was 0.28±0.82 log_10_ IU/ml (limits of agreement, −1.36 to 1.91 log_10_ IU/ml)(Figure 1B).

**Table 1:** Baseline characteristics of the 134 chronic hepatitis B patients who received nucleot(s)ide analogues therapy.

Parameter	Value
Male gender	117 (87.3)
Age (years)	45.0 (35.0–54.0)
ALT level (U/L)	34 (25.0–44.0)
Time (months), treatment initiation to enrollment	9.0 (6.0–15.0)
LAM/LdT/ETV	90 (67.2)/18 (13.4)/26 (19.4)
Positive HBeAg*	55 (45.1)
HBV DNA levels measured by TaqMan assay (log_10_ IU/ml)	1.7 (1.1–2.9)
HBV DNA levels measured by Abbott assay (log_10_ IU/ml)	1.9 (1.4–2.8)

Continuous variables were expressed as median (25^th^, 75^th^ percentile); categorical variables were expressed as number (percentage).

LAM: lamivudine; LdT: Telbivudine; ETV: entecavir;

*Missed data on 12 patients.

**Figure 2:**
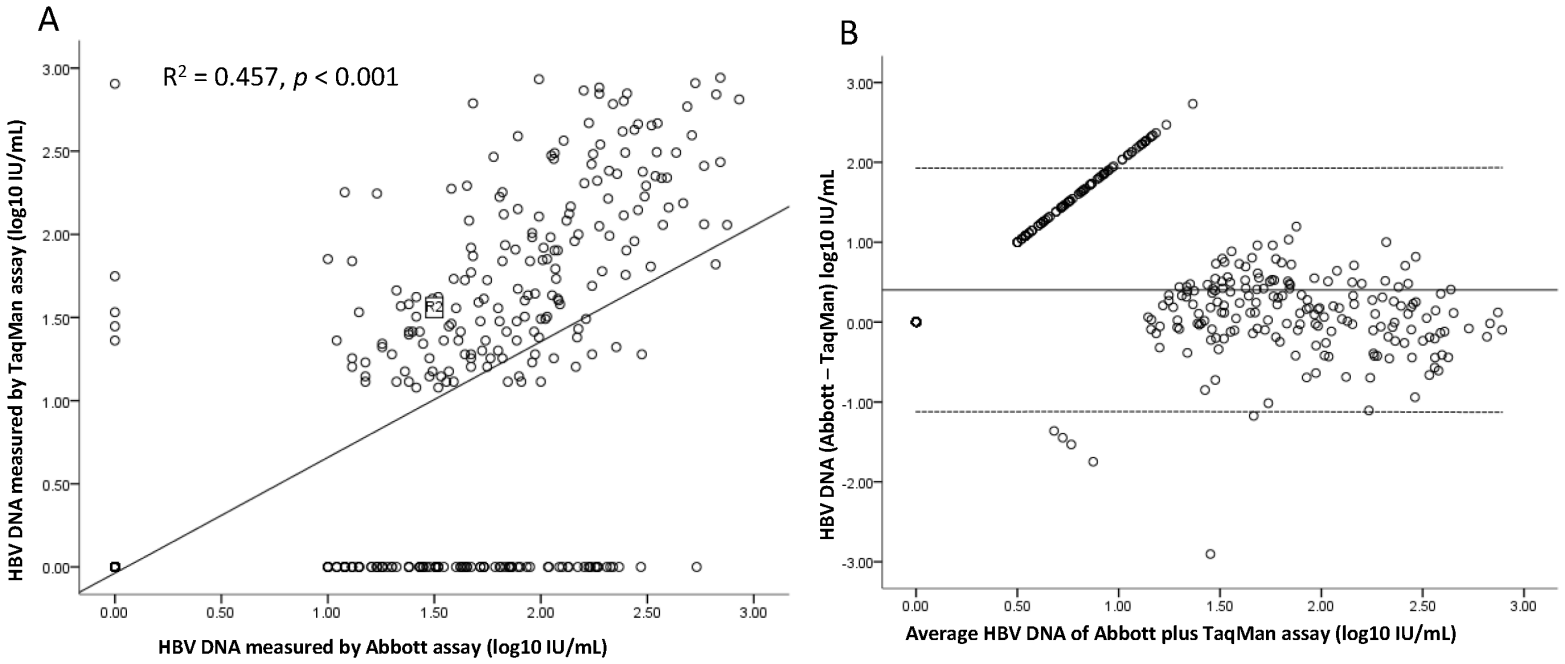
The correlation of measurements and limits of agreement between RealT*ime* and TaqMan assay in the 434 samples of HBV DNA level <3 log_10_ IU/mL. The correlation of HBV DNA in the 434 samples between the two assays was not as good as all samples (R^2^ = 0.457; *P*<0.001). (A) The mean difference was 0.40±0.77 log_10_ IU/ml (limits of agreement, −1.15 to 1.95 log_10_ IU/ml). (B)

**Figure 3:**
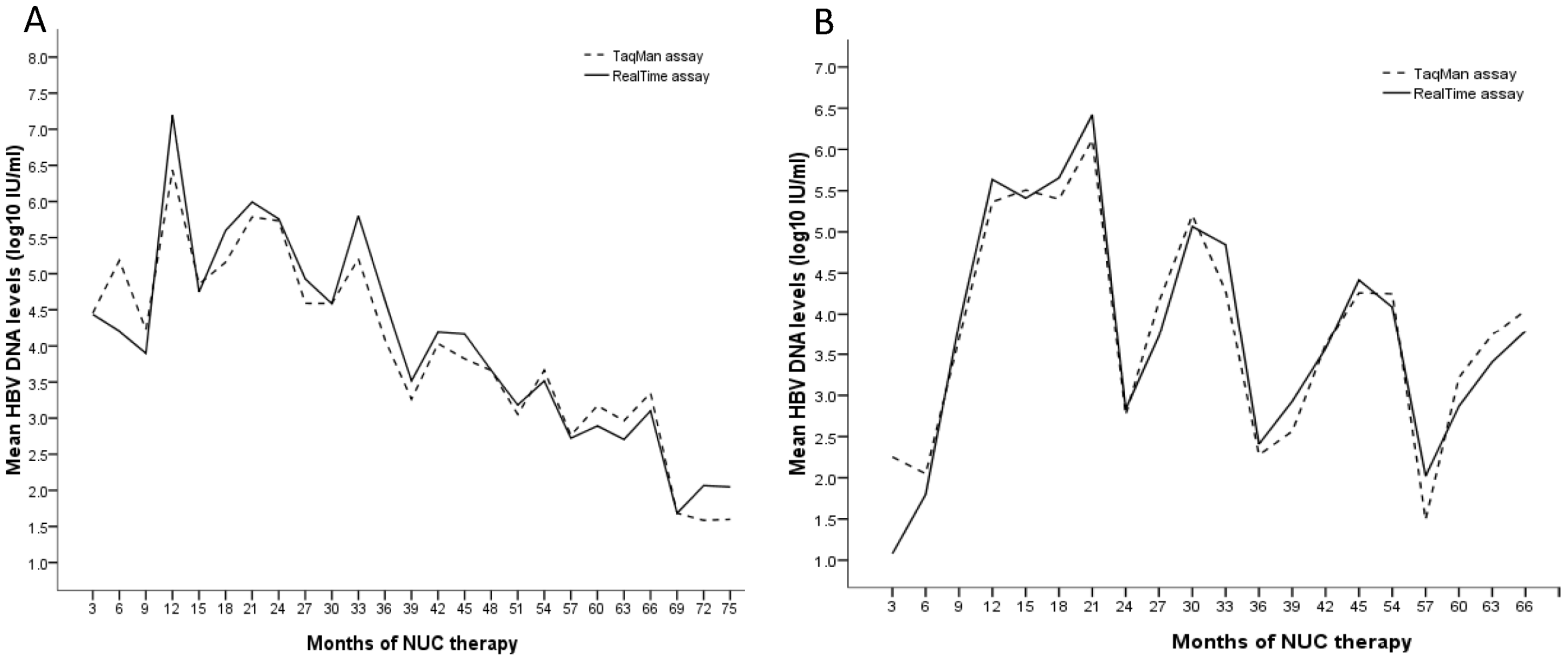
Mean HBV DNA levels of all (A) and virologic breakthrough (B) patients according to the time points of nucleot(s)ide analogues therapy. HBV DNA levels detected by *RealTime* assay were slightly higher than TaqMan assay although these differences were not statistically significant.

Among 571 serum samples, 434 (76.0%) samples had HBV DNA level <3 log_10_ IU/ml measured by both assays. The correlation between the two assays in the 434 samples was not as good as in all samples (R^2^ = 0.457, P<0.001)(Figure 2A).The mean difference was 0.40±0.77 log_10_ IU/ml (limits of agreement, −1.15 to 1.95 log_10_ IU/ml)(Figure 2B). Furthermore, 123 (21.5%) of the 571 samples had a detection difference of ≥1 log_10_ IU/ml and most (91.9%) of them had HBV DNA level <3 log_10_ IU/ml measured by both of the assays. Figure 3 showed the mean HBV DNA levels of all (A) and VBT (B) patients according to the time points of NUC therapy. Generally, HBV DNA levels detected by RealT*ime* were slightly higher than TaqMan although these differences were not statistically different. The HBV DNA level was found below lower limit of quantification in 250 and 150 samples, respectively, by TaqMan and RealT*ime* assay. Of the 250 samples below lower limit of quantification by TaqMan, 174 (69.6%) samples showed “target detected” and the remained 76 (30.4%) samples showed “target undetected”. Seventy-one (47.3%) of the 150 samples below lower limit of quantification by RealT*ime* showed “target detected” and the remained 52.7% of samples showed “target undetected”. Forty-one samples showed “target detected” by TaqMan, but “target undetected” by RealT*ime*. By contrast, 24 samples showed “target detected” by RealT*ime*, but “target undetected” by TaqMan. Notably, there were 105 samples having quantitative value by RealT*ime*, but unquantitated by TaqMan. However, only 5 samples showed contrasty results.

**Figure 4:**
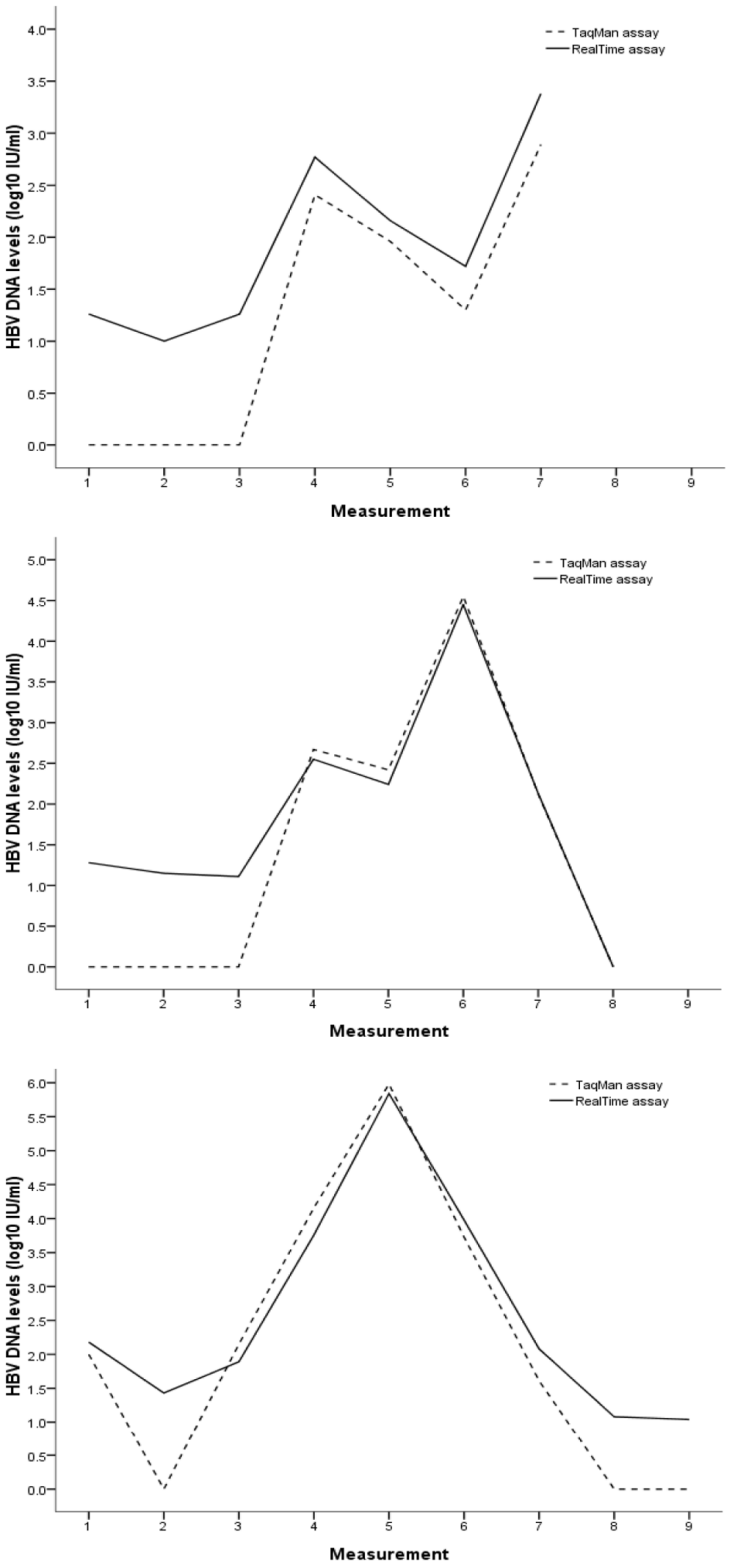
Individual HBV DNA level of the 3 patients with persist detectable DNA by RealT*ime*, but undetectable by TaqMan assay and developed YMDD mutation.

During study period, 24 (17.9%) of the patients experienced VBT. We checked YMDD mutation in all 24 patients at the time point of enrollment and VBT. YMDD mutation was not detected in any of the patients at enrollment but was found in 22 (91.7%) of the patients following VBT. We further divided the patients into 4 groups according to the status of HBV DNA detection (persistent detectable, undetectable during study period) by the two assays. For the group of undetectable HBV DNA by both assays (n = 39), no VBT was observed. Persistent detectable DNA by both assays was observed in 69 (51.5%) of all patients in this study and 21 (30.4%) of thesepatients had VBT during study period. Most patients who experienced VBT (90.5%)had accumulated the YMDD mutation. A discordant result of HBV DNA detection between the two assays was observed in 26 patients (19.4%) and of these,25 (96.2%) had persistent detectable HBV DNA by RealT*ime*, but were undetectable by TaqMan. The median (IQR) viral load of each point measured by RealT*ime* were 1.90 (0.75), 1.64 (0.64), 1.72 (0.60), 1.73 (1.06), 1.67 (0.81), 2.24 (2.07), 2.03 (0.46), 1.59 (2.14), 1.64 (0.93) log_10_ IU/mL.VBT with YMDD mutation was observed in 3 (11.6%) of these patients. Figure 4 showed the individual HBV DNA level of these 3 patients measured by the two assays every 3 months during study period. Furthermore, of the 40 patients who had undetectable HBV DNA by RealT*ime*, none experienced VBT or accumulated YMDD mutation. In contrast, there were still 3 of the 64 (4.7%) patients who had undetectable HBV DNA by TaqMan who finally had VBT with the accumulation of YMDD mutation.

## Discussion

In the present study, we demonstrated that there was a detection difference between RealT*ime* and TaqMan in viral load less than 3 log_10_ IU/ml and the detection difference may influence the identification of drug resistant mutation. Thus, RealT*ime* HBV assay may have advantages when monitoring patients receiving NUC therapy for HBV infection.

HBV viral load is currently a standard marker to assess the efficacy of HBV antiviral therapy and also a marker for the progression of liver disease [15], [16]. Sustained suppression of HBV DNA is one of the goals of HBV therapy in regional guidelines [17], [18], [19]. To achieve this goal, long term NUC therapy should be administered. The development of drug-resistance is a major concerned when placing a patient on long term NUC therapy. To facilitate the early detection of NUC drug-resistance, close monitoring of patient serum HBV DNA level is recommended. Because low level HBV viremia is associated with the early stages of developing drug resistance, using a highly sensitive real time PCR assay to detect HBV DNA level is especially important. Generally, nucleotide sequencing of the PCR product was used to detect the development of resistant mutations. However, it could detect only when the minorities of viral quasispecies were greater than 25%. A recent publication by F. Ntziora et al. reported that using a sensitive amplification refractory mutation system real-time PCR combined with molecular beacon technology could effectively detect and quantify the YMDD mutation early before emerging to dominate the population [20].

RealTi*m*e assay and TaqMan assay are two highly sensitive HBV DNA quantitative assays and are widely used commercially. In a recent short communication by Morris CJ et al. [21], they compared the two assays in detection of HBV DNA in 215 CHB patients and found that the RealTi*m*e had a wider dynamic yielding absolute quantitative values. We also observeda good correlation between the two assays among serum samples from untreated chronic hepatitis B patients, irrespective of viral status [10]. However, a discordance of ≥1 log_10_ IU/ml was also observedin 8.6% of samples and was found to be associated with HBV DNA level <3 log_10_ IU/ml. In the present study we found that 21.5% of the samples had a detection difference of ≥1 log_10_ IU/ml between the two assays and most (91.9%) of them were HBV DNA level <3 log_10_ IU/ml measured by both of the assays. The result is compatible to our previous study and the difference especially found in samples with HBV DNA < 3 log may mainly related to the different lower limit of quantification by the two assays.

Introduction of a rescue therapy as early as possible in CHB patients with VBT has been demonstrated to provide benefits in long-term clinical outcomes [22]. Previous study by Thibault V. et al. [23] demonstrated that RealTi*m*e meets the requirements for the use in monitoring response to NUC therapy and its high sensitivity is adequate for the detection of VBT at an early stage. The detection difference in low viral load may influence the detection of VBT and drug resistant mutation. In the present study, HBV DNA levels waspersistently detected in 25 (18.7%) of the 134 patients by RealT*ime* but were always undetected by TaqMan. The finding demonstrated that RealT*ime* might be more sensitive in detection of very low viral load than TaqMan. The interesting finding was not reported before.

In addition, NUCs therapy may be stopped afterat least 12 months consolidation therapy when undetectable HBV DNA is achieved in CHB patients [24]. A sensitive PCR quantitative assay is important to identify the real status of DNA suppression. In the present study, none of the patients with undetectable HBV DNA by RealT*ime* experienced a VBT compared to 4.7% of patients with undetectable HBV DNA by TaqMan who experienced VBT with the accumulation of YMDD mutation. The finding demonstrates that a proportion of patients who are considered to be achieving DNA suppression are not actually suppressed if a more sensitive HBV viral load assay is used and the risk of acquiring drug resistant mutation should be considered.

One limitation of our study is that we check YMDD mutation only in patients with VBT, not in all the patients with persistent detectable HBV DNA measured by either RealTi*m*e or TaqMan. Persistent detectable HBV DNA during NUC therapy is indicative of poor response to therapy with a high probability of developing drug-resistance. Drug-resistant mutations should also be checked in patients with persistent detectable HBV DNA. Another limitation is that the data of viral genotype is lacking. However, we believe that the majority of our patients are genotype B or C CHB. Thus, the results of the study may be drawn only on the 2 genotypes. The third limitation is that we tested the 24 patients experienced VBT for resistance mutations only in YMDD domain but not for substitutions in the region targeted by TaqMan assay. As previous mentioned that the substitution rate was higher in the HBV core gene than surface gene. Furthermore analysis of the samples that did not detect with TaqMan assay may be needed to clarify the issue.

In conclusion, these result demonstrated that Abbott RealT*ime* HBV assay is more sensitive in detection of low viral load than CobasAmpliPrep/CobasTaqMan HBV assay. The acquisition of drug-resistance mutations is a constant consideration for patients in NUC therapy. Persistently detectable low levels of HBV DNAincreases the risk of acquiring drug resistant mutationsand the use of a highly sensitive, quantitative HBV DNA assay helps in the early detection of drug resistant mutations.
